# Two functional reticulocyte binding-like (RBL) invasion ligands of zoonotic *Plasmodium knowlesi* exhibit differential adhesion to monkey and human erythrocytes

**DOI:** 10.1186/1475-2875-11-228

**Published:** 2012-07-06

**Authors:** Amma A Semenya, Tuan M Tran, Esmeralda VS Meyer, John W Barnwell, Mary R Galinski

**Affiliations:** 1 Emory Vaccine Center and Yerkes National Primate Research Center, Emory University, Atlanta, GA, USA; 2 Malaria Branch, Division of Parasitic Diseases and Malaria, Centers for Disease Control and Prevention, Atlanta, GA, USA; 3 Department of Medicine, Division of Infectious Diseases, Emory University School of Medicine, Atlanta, GA, USA; 4 Present address: Parasitic Diseases Branch, Division of Parasitic Diseases and Malaria, Centers for Disease Control and Prevention, Atlanta, GA, USA; 5 Present address: Laboratory of Immunogenetics, National Institute of Allergy and Infectious Diseases, National Institutes of Health, Rockville, MD, USA

**Keywords:** *Plasmodium knowlesi*, Malaria, Erythrocyte, Merozoite, Adhesion, Binding protein, Reticulocyte, Invasion ligand, RBLs

## Abstract

**Background:**

*Plasmodium knowlesi* is a monkey malaria species that is becoming a serious public health concern infecting hundreds and perhaps thousands of humans in Southeast Asia. Invasion of erythrocytes by merozoites entails a cascade of molecular interactions. One step involves the adhesion of *Plasmodium* reticulocyte binding-like (RBL) proteins. *Plasmodium knowlesi* merozoites express only two RBL invasion ligands, known as Normocyte Binding Proteins (PkNBPXa and PkNBPXb).

**Methods:**

Overlapping N-terminal regions of PkNBPXa and PkNBPXb were expressed in COS7 cells and tested for surface expression and adhesion to rhesus monkey erythrocytes. Subsequent tests to study specific receptor ligand interactions included adhesion to a panel of human and non-human primate erythrocytes, enzymatic treatment, and site directed mutagenesis.

**Results:**

An N-terminal cysteine-rich region of PkNBPXb (PkNBPXb-II) exhibited specific adhesion to rhesus monkey erythrocytes. Mutation of four of five cysteines in PkNBPXb-II interfered with its surface expression on COS7 cells, suggesting disulphide bond conformation is critical for intracellular trafficking. Binding of PkNBPXb-II was abolished when rhesus erythrocytes were pre-treated with chymotrypsin, but not trypsin or neuraminidase. PkNBPXb-II also bound other Old World monkey species and gibbon erythrocytes. However, erythrocytes from other primate species including humans did not bind to PkNBPXb-II or native PkNBPXb. Importantly, unlike PkNBPXb, PkNBPXa bound human erythrocytes, and this binding was independent of the Duffy blood group determinant.

**Conclusions:**

The data reported here begins to clarify the functional domains of the *P. knowlesi* RBLs. A binding domain has been identified and characterized in PkNBPXb. Notably, this study demonstrates that unlike PkNBPXb, PkNBPXa can bind to human erythrocytes, suggesting that PkNBPXa may function as a ligand to enable the invasion of *P. knowlesi* merozoites into human cells.

## Background

Malaria is an ancient disease that continues to affect several hundred million people annually in countries of Africa, Asia, and South and Central America. Four species of protozoan parasites of the genus *Plasmodium, Plasmodium falciparum, Plasmodium vivax, Plasmodium ovale* and *Plasmodium malariae*, have long been known to cause the disease with most of the nearly one million malaria-associated deaths attributable to *P. falciparum*[1]. In the last decade, a fifth species, *Plasmodium knowlesi*, a well-known cause of monkey malaria, has emerged as a potential cause of severe and fatal malaria in humans with more than 700 documented cases [2-5]; unreported cases are likely to be in the thousands in Southeast Asia.

As obligate, intracellular parasites, *Plasmodium* utilizes various strategies to efficiently gain entry into host erythrocytes and evade host immune responses, thereby achieving the ability to replicate and survive. The asexual erythrocytic stage of the *Plasmodium* life cycle is the cause of all clinical symptomology of malaria, and successful merozoite invasion is essential for the maintenance of a malaria infection and propagation of the parasites. An improved understanding of these processes is important for devising prophylactic and therapeutic strategies against the various species that cause human malaria.

*Plasmodium knowlesi* has traditionally been instrumental as a model parasite species in studies of erythrocyte invasion and has served as a stringent model for pre-clinical, proof-of-principle blood-stage malaria vaccine testing in rhesus monkeys [5-10]. Like *P. falciparum*, and in contrast to *P. vivaxP. knowlesi* merozoites are not restricted to invading reticulocytes but also invade mature erythrocytes. Merozoite invasion is a complex, multi-component process, involving a series of parasite-host molecular interactions that are not completely defined. In studies using *P. knowlesi*, after initial steps of surface adhesion, apical reorientation and host cell selection, the merozoite forms an irreversible electron-dense tight junction with the membrane of its target erythrocyte [11]. The Reticulocyte Binding Protein-Like (RBL) superfamily of ligands expressed at the apical end of merozoites has been implicated in the attachment of the apical end of this invasive stage to the surface of erythrocytes and its commitment to entering the selected host cell [10,12-14].

Two Reticulocyte Binding Proteins (RBP-1 and RBP-2), from which the RBL name is derived, were originally defined in *P. vivax* and shown to be expressed late in schizogony as large proteins (>300 kDa) that specifically bound to reticulocyte host cells [12]. Up to 10 paralogous *rbl* genes have since been identified in the *P. vivax* genome [15], a number of which have frameshift mutations or are incomplete gene fragments and may be non-functional pseudogenes (Meyer, Galinski, Barnwell, unpublished data). Based on initial RBP binding data [12] and parasite biology, the RBLs have been predicted to be critical in the initial selection and apical attachment of a merozoite to a potential host cell leading to a commitment to invasion by the subsequent release of Duffy Binding-Like/Erythrocyte Binding-Like (DBL/EBL) invasion ligand proteins from the microneme organelles and the formation of a tight junction [12,16].

A family of six paralogous *rbl* genes are now recognized in the human malaria species *P. falciparum*[17], six in the chimpanzee parasite, *Plasmodium reichenowi*[18], two in *P. knowlesi*[19,20], at least two in *Plasmodium cynomolgi* (also a simian malaria species) [19,20], and 14 in the rodent parasite *Plasmodium yoelii*[21]. The two *rbl* genes characterized so far in *P. cynomolgi* are orthologous to *rbp-1* and *rbp-2* of *P. vivax*[19]. It is likely there will be other *rbl* genes identified in *P. cynomolgi,* as in *P. vivax*, but this remains to be determined with the completion of genome sequences for this kindred species.

Studies on the RBL ligands in *P. falciparum*, also known as the reticulocyte binding protein homologs or Rh proteins, have been extensive in recent years, with these in mind as vaccine candidates [17]. Putative erythrocyte binding domains have been reported to date within the five expressed *P. falciparum* RBL proteins, PfRh1, PfRh2a, PfRh2b, PfRh4 and PfRh5 [22-26]. Genetic manipulation and invasion assays aiming to study the function of the various Rh paralogs have also given rise to the hypothesis that like the EBL invasion ligands, the RBL family may present alternative invasion pathways for *P. falciparum* parasites [27,28].

This study specifically sought to investigate the RBLs using *P. knowlesi* to better understand their role as adhesins in the intricate process of erythrocyte invasion, and their potential as a target for vaccine development. This laboratory has previously reported the identification of two intact *rbl* genes in *P. knowlesi,* and showed that they become expressed in the microneme organelles as large proteins of approximately 300 kDa; a small relic pseudogene orthologous to *rbp-1* of *P. vivax* was also confirmed in this species [20]. The *P. knowlesi* RBLs were named as normocyte binding proteins, PkNBPXa and PkNBPXb, and it was confirmed that both these proteins adhere to rhesus RBCs in traditional erythrocyte binding assays [20]. Immuno-electron microscopy further showed that the *P. knowlesi* RBLs appear to be released as merozoites attach to and invade erythrocytes [20]. Given the limited number of only two functioning *rbl* family members confirmed in the *P. knowlesi* genome, and predictably the restricted potential for alternative RBL invasion pathways, it was speculated that these proteins, and particularly the binding domains, could serve as effective immunogens for pre-clinical, proof-of-principle efficacy studies in rhesus monkeys.

In the current study, the functional adhesive characteristics of overlapping N-terminal *P. knowlesi* RBL sub-domains were explored to identify potential domains critical for ligand adhesion to erythrocytes. An erythrocyte-binding domain was identified near the N-terminus of PkNBPXb in a region with closely spaced cysteines. Importantly, native PkNBPXb did not bind to human erythrocytes, but the PkNBPXa ligand did bind to human cells. These and associated data begin to define the ligand-receptor pathways for host specificity of *P. knowlesi* in human and non-human primate hosts.

## Methods

### Cloning of *pknbpxa* and *pknbpxb g*ene segments

Segments of *pknbpxa* and *pknbpxb* were amplified using gene-specific primers and standard polymerase chain reaction (PCR) conditions (Additional file 1), and cloned into the pDisplay vector (Invitrogen, Carlsbad, CA). Positive clones were identified and sequenced using the ABI Prism BigDye Terminator Cycle Sequencing v3.1 Ready Reaction Kit (Applied Biosystems, Carlsbad, CA).

### COS7 mammalian cell line transfections

COS7 cells were cultured in DMEM containing 10 % Fetal Bovine Serum, HEPES buffer, and antibiotics at 37 °C in 5 % CO_2_. For transfections, using 1 μg of vector DNA and Lipofectamine 2000 Reagent (Invitrogen, Carlsbad, CA), the cells were plated in six-well plates at 1x10^5^ cells per well using medium without antibiotics and grown for 24 hours at 37 °C in 5 % CO_2_.

### Lysis of COS7 cells and immunoblots

Twenty-four hours after transfection, cells were lysed using CelLytic^TM^ M (Sigma-Aldrich, St. Louis, MO). Lysed cells containing expressed recombinant protein were electrophoresed on 10 % polyacrylamide gels (Bio-Rad, Hercules, CA) and transferred to nitrocellulose membranes (Schleicher & Schuell, Keene, NH). The membranes were probed with a mouse monoclonal antibody against c-myc or hemagglutinin domains (Invitrogen, Carlsbad, CA) for one hour followed by washes and incubation with the secondary antibody anti-mouse IgG conjugated to alkaline phosphatase (Promega, Fitchburg, WI) for one hour. NBT/BCIP substrate (Promega, Fitchburg, WI) was added to the membranes to detect the protein bands.

### Erythrocyte samples

Erythrocytes were obtained from fresh whole blood or cryopreserved samples. Blood from rhesus macaques, *Macaca mulatta* (Indian and Chinese origin), long-tailed macaques (*Macaca fascicularis*), pigtail macaques (*Macaca nemestrina*), sooty mangabeys (*Cercocebus atys*) and white mice was collected at the Yerkes National Primate Research Center (YNPRC) in tubes containing ACD or CPDA. Blood from owl monkeys (*Aotus nancymaae*) and squirrel monkeys (*Saimiri boliviensis*) was collected in heparinized tubes at the Centers for Disease Control and Prevention and YNPRC. Rabbit blood collected in ACD was obtained from Covance (Denver, PA). Primate blood cryopreserved in Glycerolyte 57 (Baxter Healthcare, Fenwal Div., Deerfield, IL) [29,30] from marmosets (*Callithrix jacchus*), tamarins (*Saguinus midas*), capuchins (*Cebus apella*), gibbons (*Hylobates lar*), chimpanzees (*Pan troglodytes*), and humans (*Homo sapiens*) (Duffy positive, Fy^a+b+^; and Duffy negative, Fy^a-b-^) was thawed and washed with RPMI media using standard procedures. All freshly drawn blood and thawed cryopreserved blood were stored at 4 °C for a maximum of seven days. All procedures were in accordance with protocols approved by the respective Institutional Animal Care and Use Committees.

### Immunofluorescence assays (IFA)

Temporal expression curves were developed to determine the optimal time for surface expression of each recombinant construct. Briefly, COS7 cells were incubated with mouse anti-HA antibody (Millipore, Billerica, MA) diluted 1:250 for one hour and then washed twice in DPBS. Cells were incubated with an Alexa Fluor 488 conjugated anti-mouse IgG antibody (Invitrogen, Carlsbad, CA) diluted 1:200 for one hour and then washed twice in DPBS. COS7 cells were fixed using 1 % formaldehyde for 10 minutes at room temperature. The percent of cells expressing protein on the surface was determined by counting the number of nuclei and the number of cells emitting surface fluorescence by microscopic analysis. Images were visualized using a Nikon ECLIPSE TE 300 inverted microscope with 200x magnification.

### Erythrocyte rosetting assays

COS7 cells expressing each domain were washed with complete culture media between 24 and 36 hours after transfection, when surface expression was considered optimal. The cells were incubated with rotational mixing for at least two hours at room temperature with 0.2 % erythrocytes in complete culture media. The cells were washed three times with DPBS, incubated with 1 % formaldehyde for 10 minutes at room temperature to stabilize rosettes, and then incubated with 0.1 μg/mL of Hoescht dye (Invitrogen, Carlsbad, CA) for five minutes. Erythrocyte adhesion was analysed by counting 350 cells per field in 50 fields using a Nikon ECLIPSE TE300 inverted microscope at 200X magnification. A rosette was scored as positive when most of a COS7 cell was covered by erythrocytes. The paired t-test was used to determine differences between the number of rosettes formed by the different transfectants. *P* values less than 0.05 were considered significant. Relative binding was calculated as a ratio between the number of rosette-forming erythrocytes from each host cell and the number of rosette-forming erythrocytes from rhesus cells.

### Enzymatic treatment of erythrocytes

Fifty microliters of packed rhesus erythrocytes were incubated in 1 mL RPMI with 1 mg/mL trypsin (Calbiochem, Darmstadt, Germany), 1 mg/mL chymotrypsin (Calbiochem, Darmstadt, Germany), or 0.025U/mL neuraminidase (Roche, Penzberg, Germany) for one hour at 37 °C. The cells were washed twice with RPMI and incubated with 0.5 mg/mL soybean trypsin inhibitor (Calbiochem, Darmstadt, Germany) or 1 mM phenylmethylsulfonyl fluoride, PMSF (Sigma-Aldrich, St. Louis, MO) for 15 minutes at room temperature. The cells were washed twice with RPMI and subsequently used in erythrocyte adhesion assays.

### Erythrocyte binding assays (EBAs)

*P. knowlesi* (H strain)-infected cells and supernatants were obtained from either *in vitro*-adapted culture maintained with rhesus cells or *ex vivo* from an infected rhesus macaque. *Plasmodium knowlesi* schizonts with predominantly two to four nuclei were purified by centrifugation on Percoll (Amersham, Little Chalfont, UK) gradients >95 % homogeneity [31], placed in tissue culture flasks at a concentration of 2.5 × 10^7^ parasites/mL and matured to segmented schizonts. After their rupture and release of merozoites in the absence of fresh erythrocytes, the culture media were centrifuged and supernatants containing native parasite proteins stored in liquid nitrogen. To perform EBAs, the supernatants were incubated with 1x10^9^ fresh erythrocytes, rotating at room temperature for four hours. The cells were washed twice by centrifugation through a Dow Corning 550 fluid (Dow Corning Corporation, Midland, MI) cushion. Bound proteins were eluted in 50 μl of 5x RPMI at room temperature and harvested by centrifugation at 4 °C for 10 minutes at 7,500 rpm. The eluted proteins were subjected to SDS-PAGE through 5 % gels (Bio-Rad, Hercules, CA), transferred to nitrocellulose membranes (Schleicher & Schuell, Keene, NH), and probed with rabbit polyclonal antisera to PkNBPXa or PkNBPXb for two hours followed by alkaline phosphatase conjugated anti-rabbit IgG (Promega, Fitchburg, WI) for one hour. Protein bands were visualized by adding NBT/BCIP substrate (Promega, Fitchburg, WI) to the membranes. Test rabbit antisera included anti-PkNBPXa, anti-PkNBPXb and *P. knowlesi* Merozoite Surface Protein (140 kDa) antiserum described previously [20].

### Site-directed mutagenesis

Five clones were generated with mutated cysteines (Cys^193^Gly, Cys^254^Gly, Cys^298^Gly, Cys^326^Gly, and Cys^332^Gly) using the QuickChange Multi Site Directed Mutagenesis Kit (Stratagene, La Jolla, CA). Primers were designed according to the manufacturer’s suggestion (Additional file 2). DNA from pDisplay-PkNBPXb-RII was used as a template for the mutagenic PCRs. Positive clones were identified and sequenced using a BigDye Terminator Cycle Sequencing v3.1 Ready Reaction Kit on a 3100 Genetic Analyzer (Applied Biosystems, Carlsbad, CA). The resulting sequences were analysed using MacVector^TM^ 7.2.2 (Accelrys Software Inc., San Diego, CA) to ensure the presence of mutated sites. COS7 cells were transfected with clones representing each mutation to evaluate surface fluorescence and binding to erythrocytes. A time course was developed to determine the optimal time of surface expression. The relative binding ratio was established using average data from five binding experiments.

## Results

### An N-terminal region of PkNBPXb binds to rhesus monkey erythrocytes

**Figure 1:**
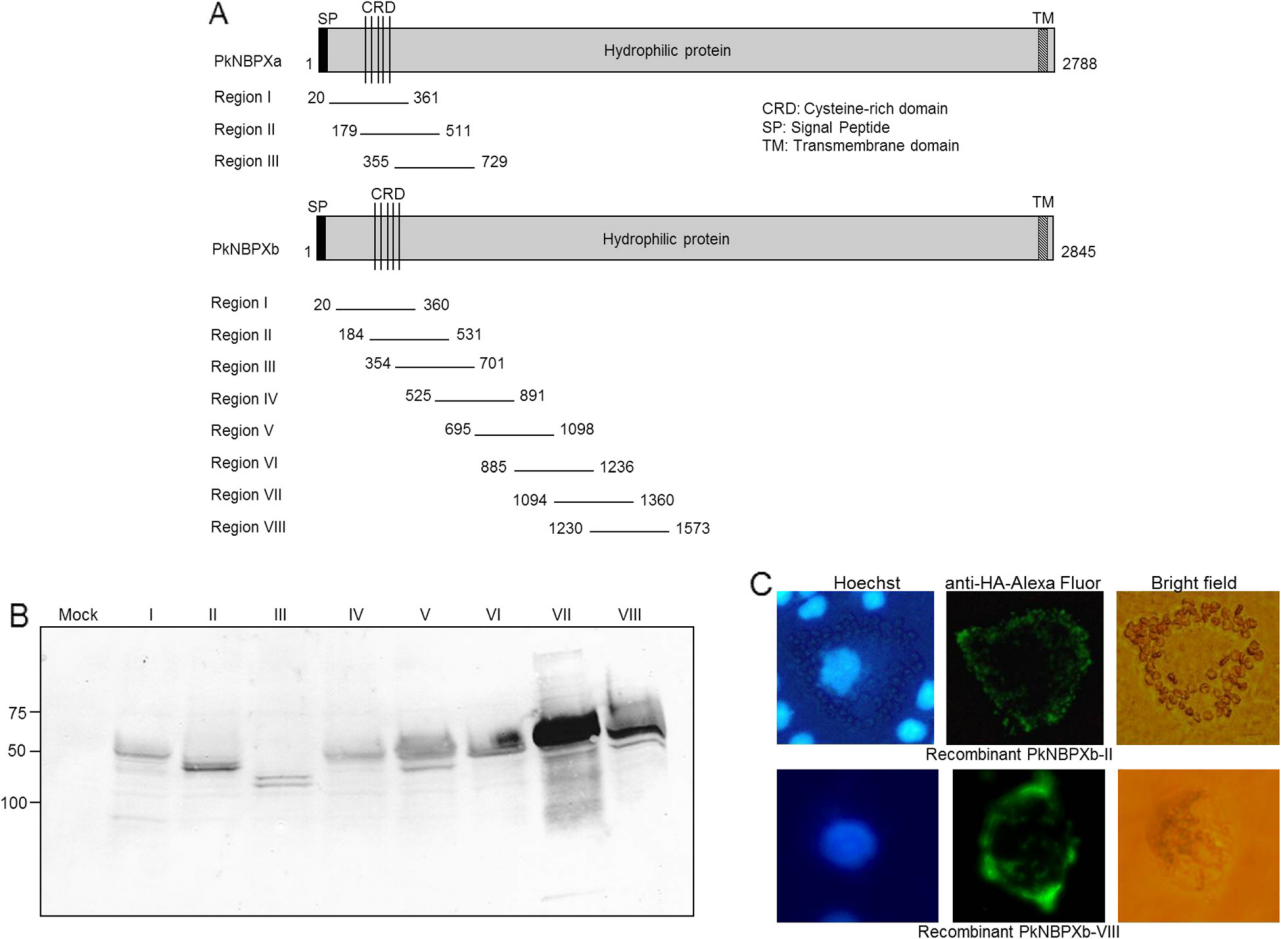
**Expression of*pknbp*gene segments in COS7 cells and binding profile to rhesus erythrocytes. (A)** Three regions spanning the 5′ end of the coding region of PkNBPXa were cloned into pDisplay as overlapping 500 nucleotide gene segments. The numbers listed correspond to the amino acid positions delineating a particular expressed gene segment. Though the recombinant proteins were expressed intracellularly, they never reached the surface of the COS7 cells (data not shown). Eight recombinant segments (Regions I - VIII) spanning the N-terminal half of PkNBPXb were cloned and expressed in COS7 cells. **(B)** Protein expression for each COS7/PkNBPXb region was evaluated by immunoblot using a specific antibody against the c-myc tag encoded in the C-terminus of the pDisplay vector. **(C)** Rhesus monkey erythrocytes were found to adhere only to PkNBPXb-II forming a large rosette pattern on the surface of COS7 cells. Panels 1 and 2 show images representing nuclear staining with Hoechst dye, COS7 cell protein expression detected by anti-HA and Alexa Fluor 488-labeled anti-mouse IgG, and erythrocyte rosetting assay results, respectively. Top and bottom panels show the surface expression and binding capability of Region II and Region VIII, respectively. Region VIII showed high expression by immunoblot while displaying no binding affinity to rhesus erythrocytes. Images were captured at room temperature using a Nikon ECLIPSE TE 300 inverted microscope using the Plan Fluor ELWD 40x / 0.45 aperture and 10x magnification eye piece and analysed using the Diagnostic Instrument, Inc. software.

Three segments from the beginning of the *P. knowlesi nbpxa* gene and eight encompassing the 5′ half of the *P. knowlesi nbpxb* gene were cloned into the pDisplay vector (Figure 1A). Each segment of approximately 1 kb, encodes about 350 amino acids, and contiguous fragments overlap by about 165 amino acids. Protein was expressed by all eight *pknbpxb* constructs (Figure 1B) and surface fluorescence was monitored using anti-HA specific antibody on intact COS7 cells. Seven of the *pknbpxb* constructs were expressed at the surface, although at different levels as judged by fluorescent and immunoblot intensities (Table 1). The one exception was Region III, which despite repeated attempts was not expressed at the surface. Surface expression was monitored by IFA approximately every three hours between 18 and 36 hours after transfections, and optimal expression was between 33 and 36 hours. The relative differences in surface expression observed correlated with the differences observed when comparing the total protein expression profiles (Table 1; Figure 1B). In contrast with the expression of different regions of PkNBPXb in pDisplay, attempts to achieve similar results with PkNBPXa clones were unsuccessful. Anti-HA specific IFAs on intact COS7 cells indicated that none of the three cloned fragments of *pknbpxa* displayed protein on the surface (data not shown), although there was internal expression of each construct in COS7 cells.

**Table 1:** Comparison of the expression of overlapping regions of PkNBPXb at the surface of COS7 cells

**Region of PkNBPXb**	**% of cells with surface protein expression (IFA – HA antibody)**	**Binding to rhesus erythrocytes**
I	1.1 % +/− 0.6 %	−
II *	3.8 % +/− 1.3 %	+
III **	0	N/A
IV	0.6 % +/− 0.4 %	−
V	1.8 % +/− 0.9 %	−
VI	1.2 % +/− 0.6 %	−
VII	7.3 % +/− 3.7 %	−
VIII	11.5 % +/− 2.2 %	−

* Average number of rosettes for region II observed in fifty fields = 93.

** Surface expression was not observed in three separate experiments for this region.

Erythrocyte adhesion (rosetting) assays were performed to test the ability of the seven distinct regions of PkNBPXb to bind rhesus erythrocytes. Five separate experiments demonstrated consistently robust rhesus erythrocyte rosette formation with Region II, while no rosettes were detected with any other regions (Table 1). The rosettes provided a clear indication of binding, with numerous erythrocytes covering the surface of the COS7 cells transfected with the *pknbpxb-II* construct. The specificity of this observation was examined by demonstrating that rhesus monkey erythrocytes adhered only to COS7 cells that specifically exhibited surface expression of the PkNBPXb-II protein (Figure 1C, panel 1). Additionally, other segments of PkNBPXb that displayed relatively higher levels of protein expression at the surface of COS7 cells did not bind any rhesus monkey erythrocytes (Figure 1C, panel 2).

### PkNBPXb-II demonstrates binding to erythrocytes from several primate species, particularly Old World monkeys and the Lesser Apes of Southeast Asia

Erythrocytes from a panel of primate species, mice, and rabbits were also tested for their ability to bind to COS7 cells expressing PkNBPXb-II, with clear distinctions (Table 2). The binding levels were averaged over several experiments and quantitatively presented in Table 2. Erythrocytes from Old World monkey species in the family Cercophithidae consistently bound to PkNBPXb-II. There were no significant differences in binding observed between rhesus erythrocytes of Indian or Chinese origin (data not shown), or between erythrocytes from *M. nemestrina*, a natural host of *P. knowlesi.* However, erythrocytes from the primary natural host, *M. fascicularis* (*p* < 0.01), and the sooty mangabey monkey (*p* < 0.001) bound PkNBPXb-II expressing COS7 cells on average 4.3 times higher than rhesus monkey erythrocytes (Table 2). Surprisingly, PkNBPXb-II expressing COS7 cells also demonstrated rosette formation with erythrocytes from gibbons, a Lesser Ape from Southeast Asia, which was two-fold greater than for rhesus erythrocytes. This increase in binding of gibbon compared to rhesus erythrocytes is statistically significant (*p* < 0.02). Erythrocytes from humans and chimpanzees did not adhere and form rosettes in these assays. Erythrocytes from New World primate species either bound weakly (tamarins) or showed no binding (owl, squirrel, marmoset and capuchin monkeys). Relatively weak binding was also observed when COS7 cells expressing PkNBPXb-II were tested in the rosetting assays using rabbit erythrocytes, but mouse cells did not bind.

**Table 2:** Binding profile of erythrocytes from various primate and non-primate species to PkNBPXb-II expressing COS7 cells

**Common Name**	**Scientific Name**	**Regional Classification**	**Geographical Range**	RBC* Rosettes	**rPkNBPXb-II Binding**
Rhesus Macaque	*Macaca mulatta*	Old World	India, China, Thailand	21.2+/−17.6	Yes
Long-tailed Macaque	*Macaca fascicularis*	Old World	Southeast Asia	87.5+/−31.6	Yes
Pigtail Macaque	*Macaca nemestrina*	Old World	Southeast Asia	19.0+/−7.8	Yes
Sooty Mangabey	*Cercocebus atys*	Old World	West Africa	85.0+/−42.7	Yes
Owl Monkey	*Aotus nancymaae*	New World	South America	0.0	No
Squirrel Monkey	*Saimiri boliviensis*	New World	South America	0.0	No
Capuchin	*Cebus apella*	New World	South America	0.0	No
Marmoset	*Callithrix jaccus*	New World	South America	0.0	No
Tamarin	*Saguinus midas*	New World	South America	2.4+/−2.6	weak
Human	*Homo sapiens*	Great Apes	Worldwide	0.0	No
Chimpanzee	*Pan troglodytes*	Great Apes	West and Central Africa	0.0	No
Gibbon	*Hylobates lar*	Lesser Apes	Southeast Asia	49.6+/−31.4	Yes
Rabbit	*Oryctolagus cuniculus*	Rabbit		4.1+/−6.6	weak
Mouse	*Mus musculus*	Mouse		0.0	No

*Mean number and standard deviation of COS7 cells with surface bound rosettes of erythrocytes per 50 fields of 40x objective.

### Chymotryspin treatment of rhesus erythrocytes prevents adhesion to PkNBPXb-II, but not the native protein, PkNBPXb

**Figure 2:**
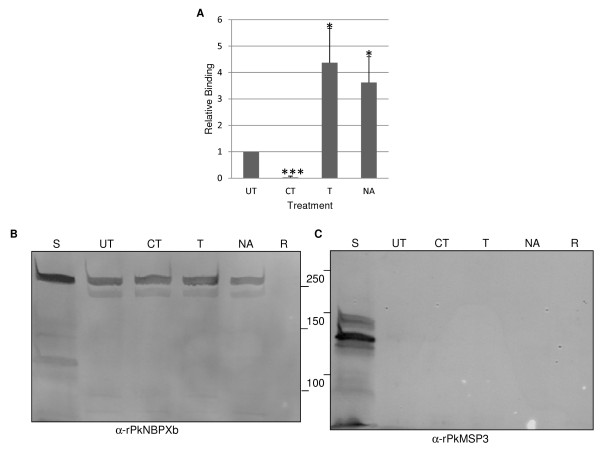
**Binding of PkNBPXb-II and native PkNBPXb to enzymatically treated rhesus monkey erythrocytes.** (**A**) PkNBPXb-II cells were tested for relative binding in adhesion (rosetting) assays to rhesus monkey erythrocytes that were either untreated, or treated with chymotrypsin (CT), trypsin (T), or neuraminidase (NA), * *p*-value <0.05 and *** *p*-value <0.001. (**B**) Native PkNBPXb binding was tested in erythrocyte binding assays with rhesus monkey erythrocytes that were untreated or treated with CT, T, or NA. Total supernatants (TS) and comparable eluates from untreated rhesus monkey erythrocytes (R) incubated without supernatants were also applied to the SDS-PAGE gels and transferred to nitrocellulose membranes. The immunoblot was probed with an anti-PkNBPXb antibody [20]. (**C**) A negative binding control was included using duplicate aliquots of the RBC samples tested in B whereby anti-PkMSP3/140 antibody recognizes the native protein in the supernatant only.

Rhesus monkey erythrocytes treated with chymotrypsin, neuraminidase, or trypsin were tested in the erythrocyte adhesion (rosetting) assays to determine basic features of the receptor(s) involved in the interaction of both, native PkNBPXb and COS7-expressed PkNBPXb-II with erythrocytes. Pre-treatment of the erythrocytes with chymotrypsin nearly abrogated binding of the erythrocytes to PkNBPXb-II (Figure 2A). The >90 % decrease in binding of chymotrypsin-treated erythrocytes is statistically significant (*p* < 0.001) compared to untreated erythrocytes. In contrast, cells expressing PkNBPXb-II bound trypsin and neuraminidase-treated erythrocytes greater than erythrocytes that were not enzymatically treated. This increased binding of trypsin and neuraminidase-treated erythrocytes is significantly greater (*p* < 0.05) than binding of untreated erythrocytes.

The chymotrypsin-sensitive profile of PkNBPXb-II adhesion contrasts with the binding profile of the largely intact native PkNBPXb to rhesus monkey erythrocytes in EBAs using *P. knowlesi* culture supernatants (Figure 2B). Rhesus erythrocytes were treated with neuraminidase, trypsin, or chymotrypsin, and eluates from EBAs using these cells were probed in immunoblot assays with a rabbit anti-PkNBPXb specific antibody [20]. Protein bands >250 kDa, corresponding to the full-length protein were observed in all samples that were incubated with the culture supernatant (Figure 2B). Interestingly, after testing supernatants in several experiments, the immunoblot signal on neuraminidase-treated and untreated rhesus cells incubated with the native PkNBPXb was consistently stronger than trypsin- or chymotrypsin-treated rhesus cells (not shown). No protein bands were detected with untreated rhesus erythrocytes incubated in the absence of culture supernatants. As a control to judge the specificity of adhesion of these cells to native PkNBPXb, the same samples were also probed with an antibody against an abundant merozoite surface protein (PkMSP3_140_) (Figure 2C). PkMSP3_140_ is known to be present in culture supernatants, but it does not adhere to the surface of rhesus erythrocytes [20]. When the samples were probed with the rabbit anti-PkMSP3_140_ antibody, the native protein was only detected in the supernatants.

### Four cysteine residues within PkNBPXb-II are critical for surface expression on COS7 cells

**Figure 3:**
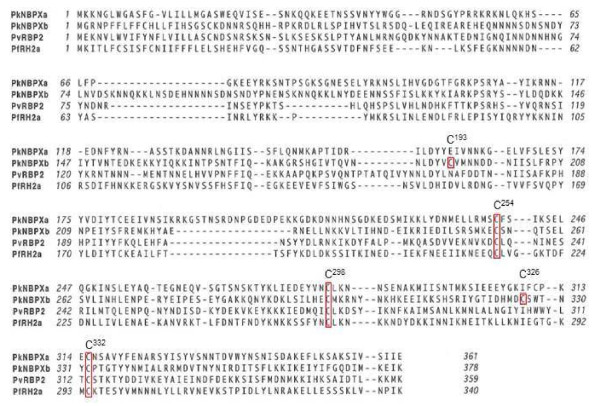
**Amino acid alignment of the N-terminal regions of PkNBPXa, PkNBPXb, PvRBP2 and PfNBP2a.** Alignment of the first 300–400 amino acids of the N-terminus of PkNBPXa, PkNBPXb, PvRBP2, and PfNBP2a demonstrates conservation of three cysteine residues among species. These three residues in PkNBPXb correspond to Cys^254^, Cys^298^, and Cys^332^. Two other cysteine residues are also present in the N-terminus of PkNBPXb-II and are Cys^193^ and Cys^326^.

The PkNBPXb-II recombinant protein contains five cysteine residues (Cys^193^, Cys^254^, Cys^298^, Cys^326^, Cys^332^), which were hypothesized to be critical in the formation of the correct tertiary conformation of the binding domain. Alignment of the first 300–400 amino acids of PkNBPXb-II, PkNBPXa, and the related RBL proteins in *P. vivax* (RBP2) and *P. falciparum* (Rh2a/b) demonstrated conservation of three of these cysteines (Figure 3).

**Figure 4:**
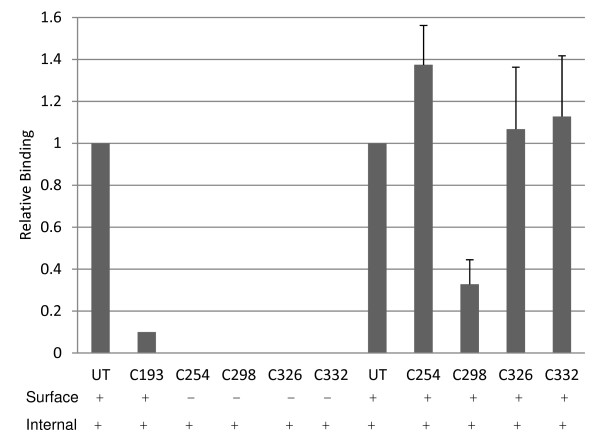
**Four disulphide bonds in PkNBPXb are important for expression on the surface of COS cells.** The N-terminal domain of PkNBPXb contains five cysteine residues (C^193^, C^254^, C^298^, C^326^, C^332^). Using site directed mutagenesis, all five residues were individually mutated to glycine and all but C^193^ were found to be required for binding to erythrocytes. The glycine residues present in each construct were mutated back to cysteine residues; these constructs were able to express protein on the surface and also able to bind erythrocytes. Surface and internal expression is indicated with + or – signs.

To determine which of these cysteines may be important for the formation of disulphide bonds and if they are required for the adhesion of erythrocytes, site-directed mutagenesis was performed to replace each of them with glycine residues. Five mutated *pknbpxb*-*II* pDisplay constructs, confirmed by DNA sequencing, were tested for protein surface expression every three hours from 18–36 hours after transfection. When Cys^193^ was mutated, protein surface expression was observed, albeit at reduced levels. Rosetting of erythrocytes was also observed but again at reduced levels (~90 %) (Figure 4). However, protein surface expression was not observed for the other four mutated *pknbpxb*-*II* constructs (Cys^254^, Cys ^298^, Cys^326^, Cys^336^), and, as a consequence, rosettes were not observed with cells transfected with these constructs (Figure 4B). However, when these mutated constructs were genetically manipulated again to encode cysteine in place of the glycine residues, protein surface expression and erythrocyte binding were restored (Figure 4).

### Native PkNBPXa binds to human erythrocytes

**Figure 5:**
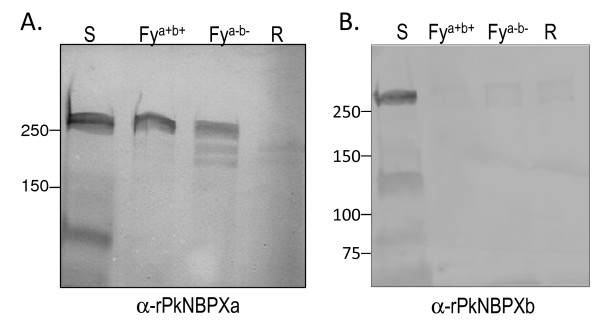
**PkNBPXa binds to Duffy negative and Duffy positive human erythrocytes.** Native PkNBPXa (**A**) and PkNBPXb (**B**) present in parasite supernatants were tested for their ability to bind Duffy positive and Duffy negative human cells by EBA. Culture supernatants (S) containing soluble merozoite proteins were incubated with human Duffy positive (Fy^a+b+^) and Duffy negative (Fy^a-b-^) erythrocytes, and then the bound proteins were eluted, electrophoresed and transferred to nitrocellulose membranes. PkNBPXa and PkNBPXb specific antibodies were used to probe the membranes. PkNBPXa was detected in the supernatants and eluates from Duffy positive and Duffy negative erythrocytes. PkNBPXb was present in the supernatant but did not bind to either type of human erythrocytes. As a control, erythrocytes were incubated in the absence of supernatants and no PkNBPXa specific bands were detected in those samples (R).

The results above indicated that COS7 cells expressing PkNBPXb-II do not form rosettes with human erythrocytes, going against what might be expected since *P. knowlesi* is known to infect and cause malaria in humans [4,5]. This study therefore investigated whether either or both of the native proteins, PkNBPXa and PkNBPXb, would bind to human erythrocytes in standard EBAs [20]. Both Duffy positive (Fy^a+b+^) and negative (Fy^a-b-^) human erythrocytes were incubated with *P. knowlesi* culture supernatants and the bound proteins were eluted, processed through SDS-PAGE, blotted, and probed with antibodies specific to either PkNBPXa or PkNBPXb [20]. This assay showed that only native PkNBPXa bound human erythrocytes (Figure 5). As a negative control, and to support the binding specificity of PkNBPXa, the same samples were probed with rabbit antiserum against PkMSP3_140_. Samples containing culture supernatants alone reacted with the PkMSP3_140_ antibodies, but not the eluates from EBAs (data not shown). The inability of the native PkNBPXb to bind human erythrocytes in these assays is consistent with the data shown here demonstrating that the PkNBPXb-II expressed at the surface of COS7 cells did not bind to human erythrocytes. These experiments also demonstrated that the native PkNBPXa protein was able to bind to both Duffy positive and Duffy negative RBCs in the erythrocyte binding assays with similar intensities.

## Discussion

Through COS7 cell surface expression and rosetting adhesion assays, this study has identified an erythrocyte-binding domain within the N-terminus of one of two *P. knowlesi* RBL proteins, namely PkNBPXb. This binding domain wase called PkNBPXb-II, because it was the second in a series of eight overlapping protein constructs tested. Using similar procedures, a comparable domain could not be identified near the N-terminus of PkNBPXa. Importantly, however, the only other RBL ligand expressed by *P. knowlesi* merozoites, PkNBPXa, in contrast with PkNBPXb, strongly binds human erythrocytes in addition to monkey host erythrocytes by traditional EBAs.

This study focused on the N-terminal half of the PkNBPs [20] given certain intuitive considerations about RBL structure and the exposure of binding sites, as well as previous studies characterizing the binding domain of PvRBP1 [32]. In addition, erythrocyte-binding domains of *P. falciparum* and *P. yoelii* RBL family members have since been identified in various N-terminally associated regions [22,23,33].

PkNBPXb-II was the only domain found to bind erythrocytes. This domain includes amino acids 184 to 531, contains five cysteines, and resides in the relatively cysteine-rich N-terminal area designated as the Rh homology region in RBL paralogs in *P. falciparum*[23,25,34]. Interestingly, this region of low homology was first delineated by clustal alignment of *P. vivax* RBP1 with Rh1 and Rh4 [23]. The binding domains for PfRH4 and PfRH5 appear to reside in this zone of weak homology based on the binding of recombinant peptides designed from this region [23,25]. However, the binding domains for two other *P. falciparum* family members, Rh1 and Rh2a/Rh2b, apparently reside just outside this region of Rh homology [22,24,26].

The RBL invasion ligand proteins contain a variable number of cysteines in the N-terminal region of homology, but it has not been known if these residues participate in disulphide bond conformation necessary for receptor-ligand binding interactions. This study shows that the mutation of four individual cysteine residues prevented the trafficking to and expression of the PkNBPXb-II protein on the surface of COS7 cells, suggesting that cell-surface expression of PkNBPXb-II is dependent on critical cysteine residues. Only the mutation of Cys^193^ to Gly did not completely abolish the binding of NBPXb-II to erythrocytes, but expression was reduced along with a reduction of binding levels by 85 or 90 % as compared to cells expressing unaltered NBPXb-II. Taken together the data suggest that cell-surface expression of PkNBPXb-II is dependent on critical cysteine residues and this lack of trafficking to the surface could indicate that disulphide formation is a functionally important feature in this region of the protein. Mutagenesis of amino acid residues has been performed to map *Plasmodium* blood-stage parasite binding domains in PfEMP1DBLβ-C2 [35] and PvDBP [36,37]. Although functionally important cysteine-rich regions have been predicted within the N-terminal regions of PfRH1, PfRH2a/2b, PfRH4 and Py235 [23,24,33,38,39], there have been no studies conducted, with mutations or otherwise, to show cysteine functionality in these ligands. The RBL N-terminal regions share at least three conserved cysteines (Figure 3), and based on this data it is reasonable to hypothesize that they may be important for the conformational dependent functions of some or all of the RBLs.

Different enzymatic treatments of erythrocyte target cells can be useful to generate receptor profiles and delineate potential receptors for particular binding proteins. This study shows that chymotrypsin treatment of rhesus erythrocytes abolished the formation of erythrocyte rosettes with PkNBPXb-II, but not trypsin or neuraminidase treatments. In contrast, adhesion of native PkNBPXb to treated or untreated rhesus erythrocytes in EBAs was positive, although binding was consistently stronger with neuraminidase-treated or untreated cells. These data indicate that PkNBPXb-II contains a binding domain when tested in rosetting assays, yet the native protein may have additional co-functional domains resistant to enzyme treatment. Recently, ligand-binding specificity has been studied in detail, including enzymatic cleavage profiles in PfRh2a/b a homolog of the PkNBPs that suggest more than one binding domain in a RBL invasion ligand [40].

Native PkNBPXb as well as the PkNBPXb-II domain also has a host specific target cell binding preference. Native PkNBPXb in EBAs bound to rhesus monkey erythrocytes, but not to human erythrocytes, a result similar to that of rosetting assays with PkNBPXb-II. PkNBPXb-II robustly bound rhesus erythrocytes and even better when the rosetting erythrocytes were from the primary monkey host, *M. fascicularis* or mangabey and gibbon species. However, this binding domain and native PkNBPXb did not bind erythrocytes from humans, chimpanzees and for the most part New World Monkeys.

*Plasmodium knowlesi* has emerged as an important zoonotic human pathogen of increasing public health significance [1,2,5,41]. A future focus on studying PkNBPXa adhesion will be important because, in contrast to PkNBPXb, PkNBPXa not only binds to rhesus erythrocytes in EBA assays [20], but also human erythrocytes (Figure 5). This study attempted to identify a binding domain in PkNBPXa. However, while seven segments of PkNBPXb were readily cloned and expressed at the surface of COS7 cells, comparable cloned segments of PkNBPXa that are surface expressed have not yet been developed, despite many attempts. Future attempts to optimize both expression and trafficking of PkNBPXa regions may include the adjustment of the boundaries of these regions or changes in the expression vector, mammalian host cells, or expression conditions. Expanded studies involving PkNBPXa adhesion to human and other non-human primate erythrocytes will hopefully elucidate the role of this protein, perhaps as the RBL ligand used by *P. knowlesi* to naturally infect humans.

Interestingly, the host origin of erythrocytes correlates to some degree with the binding specificities observed, regardless of the actual ability of *P. knowlesi* to invade a particular primate cell type. For example, PkNBPXb binds strongly to erythrocytes from Old World monkeys (long-tailed, rhesus and pigtail macaques) with a Southeast Asian or African origin (sooty mangabeys) and to Lesser Apes (gibbons), which also originate in Southeast Asia, but not to erythrocytes from humans (or chimpanzees) or New World primates. Rosetting of macaque erythrocytes was expected because rhesus macaques are utilized as an experimental model studying *P. knowlesi* infections*,* and pigtail and long-tailed macaques are the natural hosts for *P. knowlesi*. Less expected, because human and chimp erythrocytes did not bind, was the strong binding observed to gibbon erythrocytes. Gibbons are neither natural nor normally experimental hosts for *P. knowlesi*, but can be infected with this parasite. Although New World monkeys, squirrel, owl and marmoset monkeys are known to be very susceptible to *P. knowlesi* infection [42-44], erythrocytes from these species did not form rosettes with PkNBPXb-II. Similarly, and importantly, human erythrocytes did not bind PkNBPXb-II or native NBPXb, even though *P. knowlesi* does infect humans. Since PkNBPXa not only binds erythrocytes from Old World monkeys, but also erythrocytes from humans, this RBL ligand may play an important role in allowing *P. knowlesi* to infect this host. These observations are consistent with the necessity of multiple receptor-ligand interactions being important to achieve successful invasion of erythrocytes from a wide range of hosts.

## Conclusions

This study has provided comparative data relevant for understanding how RBLs interact with host erythrocytes. It is well-established that *P. knowlesi* (H strain) infection in rhesus macaques is a lethal infection unless treated with anti-malarial drugs, but only recently has its clinical significance in causing mild to severe and lethal infections in humans been appreciated [2-5,41,45]. The continued investigation of *P. knowlesi* in rhesus and other macaques is now of direct relevance to humans. The identification of the PkNBPXb-II erythrocyte binding domain and differential binding specificities between PkNBPXa and PkNBPXb are important steps towards better understanding the role(s) of the RBL family of merozoite invasion ligands in host selection and advancing pre-clinical vaccine trials in rhesus macaques to determine whether RBL domains can in fact elicit efficacious protective immune responses.

## Competing interests

The authors declare that they have no competing interests.

## Authors’ contributions

AAS designed the study, performed experiments, analysed and interpreted results, and wrote the manuscript. TMT contributed to the research design, discussion of data and manuscript writing. EM generated reagents, contributed to experimental design, interpreted results and contributed to manuscript writing. JWB generated reagents, contributed to data analysis, concept discussions and manuscript writing. MRG contributed to study design, analysis, interpretation of results, and manuscript writing. All authors read and approved the final manuscript.

## Supplementary Material

Additional file 1 Primer sequences for regions cloned into pDisplay vector. Click here for file

Additional file 2 Primer sequences for site-directed mutagenesis of PkNBPXb-II. Click here for file
