# Supplementary material for: Two functional reticulocyte binding-like (RBL) invasion ligands of zoonotic Plasmodium knowlesi exhibit differential adhesion to monkey and human erythrocytes
Source: Malar J. 2012 Jul 6;11:228. doi: 10.1186/1475-2875-11-228 (PMC3464698; doi:10.1186/1475-2875-11-228)
Supplement: Additional file 1 — Primer sequences for regions cloned into pDisplay vector. [file 1475-2875-11-228-S1.pdf]

Additional File 1. Primer sequences for regions cloned into pDisplay vector

Gene	Region	Primer Sequence
pknbpxb	I (pDisplay)	Sense: 5’-ccccagatctTCATGTAAGGACAATAATAG-3’<br>Antisense: 5’-ccccgtcgacTAGAAATGACGTAATATCTC-3’
	II (pDisplay)	Sense: 5’-ccccagatctACACAAGTGAATAATTTGGA-3’<br>Antisense: 5’-ccccgtcgacATTGGAATAAAGAATATGTTC-3’
	III (pDisplay)	Sense: 5’-ccccagatctAGAGATATTACGTCATTTCT-3’<br>Antisense: 5’-ccccgtcgacGTATTCTTTTATCAACGTGT-3’
	IV (pDisplay)	Sense: 5’-ccccagatctGAACATATTCTTTATTCCAAT-3'<br>Antisense: 5’-ccccgtcgacGGTACTTTCCTTATTAACAA-3'
	V (pDisplay)	Sense: 5’-ccccagatctGACACGTTGATAAAAGAATA-3'<br>Antisense: 5’-ccccgtcgacGTCCACAAGGGATGATTT-3'
	VI (pDisplay)	Sense: 5’-ccccagatctGTTGTTAATAAGGAAAGTAC-3'<br>Antisense: 5’-ccccgtcgacGAACATTTCCATATACGAAT-3'
	VII (pDisplay)	Sense: 5’-ccccagatctTCATCCCTTGTGGACATG-3'<br>Antisense: 5’-ccccgtcgacGTAGACATCTTCTGTTCC-3'
	VIII (pDisplay)	Sense: 5’-ccccagatctGATTCGTATATGGAAATGTT-3'<br>Antisense: 5’-ccccgtcgacGTCCACATTATTAGAATTATT-3'
