# Supplementary material for: Two functional reticulocyte binding-like (RBL) invasion ligands of zoonotic Plasmodium knowlesi exhibit differential adhesion to monkey and human erythrocytes
Source: Malar J. 2012 Jul 6;11:228. doi: 10.1186/1475-2875-11-228 (PMC3464698; doi:10.1186/1475-2875-11-228)
Supplement: Additional file 2 — Primer sequences for site-directed mutagenesis of PkNBPXb-II. [file 1475-2875-11-228-S2.pdf]

Additional File 2. Primer sequences for site-directed mutagenesis of PkNBPXb-II

Gene	Cysteine	Primer Sequence
pknbpxb-II	First Cysteine	MPC1: 5’ GGATTATGTTGGTGTCATGAATAATGATGATAATATAATTTCTTTATTCCG 3’
	Second Cysteine	MPC2: 5’ TCGGAGTATGAAGGAAGGCTCCAATCAGACTTCTG 3’
	Third Cysteine	MPC3: 5’ GTATTTTACATGAAGGCATGAAAAGAAATTATAACAAACATAAAGAGG 3’
	Fourth Cysteine	MPC4: 5’ ATAGATCATATGGATGGCAGCTGGACTAACTACTGTCC 3’
	Fifth Cysteine	MPC5: 5’ TGCAGCTGGACTAACTACGGTCCAACAGGTACATATTAC 3’
	Second Cysteine Reversion Mutation	MPCR2: 5’ TCGGAGTATGAAGGAATGCTCCAATCAGACTTCTG 3’
	Third Cysteine Reversion Mutation	MPCR3: 5’ GTATTTTACATGAATGCATGAAAAGAAATTATAACAAACATAAAGAGG 3’
	Fourth Cysteine Reversion Mutation	MPCR4: 5’ ATAGATCATATGGATTGCAGCTGGACTAACTACTGTCC 3’
	Fifth Cysteine Reversion Mutation	MPCR5: 5’ TGCAGCTGGACTAACTACTGTCCAACAGGTACATATTAC 3’
